# The enrolment gap: who is not enrolling with primary health organizations in Aotearoa New Zealand and what are the implications? An exploration of 2015–2019 administrative data

**DOI:** 10.1186/s12939-021-01423-4

**Published:** 2021-04-06

**Authors:** Maite Irurzun-Lopez, Mona Jeffreys, Jacqueline Cumming

**Affiliations:** Health Services Research Centre, Faculty of Health l Te Wāhanga Tātai Hauora, Te Herenga Waka- Victoria University of Wellington, PO Box 600, Wellington, 6140 New Zealand

**Keywords:** Primary health care, Patient enrolment, Health equity, Primary health organization, New Zealand

## Abstract

**Background:**

Primary Health Care (PHC) is the entry point to accessing health services in many countries. Having a high proportion of the population enrolled with a PHC provider is key to ensuring PHC fulfils this role and that it contributes to achieving better equity in health. We aimed to understand the extent to which people in Aotearoa New Zealand are enrolling with Primary Health Organizations (PHOs), how enrolment rates have evolved over time, and variations across District Health Boards (DHBs) and socio-demographic groups.

**Methods:**

We analysed administrative data on the proportion of people enrolled in PHOs and breakdowns across DHBs, and by age, ethnicity and deprivation, for the years 2015–2019.

**Results:**

About 6% of the population was not enrolled in 2019. There are persistent differences across socio-demographic groups as well as geographically. Māori have lower enrolment rates than New Zealand European/Other groups. Young people (15–24 years) are the least likely to be enrolled. The most affluent areas have the highest enrolment rates. Auckland DHB shows the lowest enrolment rates.

**Conclusions:**

Enrolments remain below full population coverage and inequities exist between socio-demographic and geographic groups. Potential reasons explaining these trends include methodological limitations as well as real issues in accessing services. We recommend (a) work towards minimising data issues in relation to this indicator to improve its accuracy and value in signalling trends in access to PHC services, and (b) investigating the reasons for the potential widening of the inequities identified, in particular issues preventing Māori and younger people from enrolling. This study deepens our understanding of enrolment rates as an indicator for tracking equity in PHC. Other countries can learn from the Aotearoa New Zealand case to draw lessons for improving equity in health care.

**Supplementary Information:**

The online version contains supplementary material available at 10.1186/s12939-021-01423-4.

## Background

Primary Health Care (PHC) is the entry point to accessing health services in many countries. PHC embraces essential services, is typically embedded in local communities, and should be provided at an affordable cost [1]. Not only does PHC help improve population health, better access to and use of PHC is also associated with improved health equity [2]. PHC also plays a gatekeeper role in preventing and reducing the need for more costly specialized care and hospitalizations, thus having the potential to lower overall health expenditures [3].

Some countries like Italy, Netherlands, Aotearoa New Zealand, Norway, Portugal, Spain, and the United Kingdom have chosen to formalise the relationship between the PHC provider and the population through an enrolment system, also called registration or patient list. Registering with a primary care provider where the provider serves as the focal point for co-ordinating care, is mandatory in 14 countries of the Organisation for Economic Co-operation and Development (OECD)^1^ and encouraged in 10 others,^2^ out of 26 surveyed [3]. The design of the enrolment system differs across countries. For example, patient enrolment can take place with a General Practitioner (GP), practice, primary care organization, local government or insurance company [4]. Enrolment can also be voluntary with patients choosing their own GP (e.g. Aotearoa New Zealand and Norway), or compulsory where a patient is assigned to a GP or practice, usually by geographic area (e.g. the Netherlands) with some possibility of changing the allocated affiliation [4, 5]. For the patient, formal enrolment might mean an obligation or commitment to use the provider with whom they are enrolled as their preferred service. Benefits for patients who enrol vary by country and can include, for example, lower costs of consultations (Aotearoa New Zealand), guaranteed access to GPs and access to afterhours services (Ontario, Canada), and priority access to GPs (Norway). The registration system often sits alongside and has synergies with a capitation scheme that funds providers according to the number and characteristics of their enrolled population [5].

It is internationally recognised that having the population enrolled with PHC providers has many benefits favourable towards PHC values [4–7]. First, enrolments, along with capitation funding, allow a shift in provider responsibility from just treating sick patients to being responsible for and actively promoting health for a well-defined population. Second, through its associated incentives for both providers and users (e.g. lower consultation fees for patients and capitation payments for practices) it promotes early access. Third, enrolment enhances continuity of care with the same provider by formally linking up patients to a specific provider and establishing a provider’s responsibilities to be pro-active with respect to health promotion (e.g., for regular screening). Enrolment can further enhance continuity of care by formalising the PHC provider’s role in coordinating information and care for the enrolled population (e.g., with GPs taking responsibility for referral processes and follow-up). Fourth, because the enrolment system designates one place - the main provider - where a patient’s information is stored and managed, it promotes information coordination. This synchronization serves to provide an overview of all that is happening with a patient’s health, supporting better diagnosis and care. Fifth, enrolment supports health planning by allowing health providers to target health services to meet the enrolled population’s health needs. Sixth, enrolment brings gains in value-for-money across a health system, as it enhances access to and continuity of PHC, and supports the PHC gatekeeping role, thus reducing the need for more costly specialized care [5]. It also brings economic gains through the efficiencies associated with coordination of information, and by encouraging cost-effective prevention and health promotion. Seventh, regarding equity, enrolment may support equal access by reaching marginal groups and supporting economically disadvantaged users through economic incentives such as lower costs of services for those enrolled compared to those not enrolled. An enrolment system may also be beneficial for equity when it includes complementary measures to promote public trust and reaching out to those usually left out of the system. On the other hand, it may decrease equity when providers may want to avoid registering high need patients if they are not sufficiently compensated for meeting their needs. Finally, enrolment is likely to bring about accountability gains. With an enrolment system, the contracts between PHC providers and the funding agency define the nature and extent of the responsibility of providers towards their enrolled population regarding health promotion, ongoing care, information management, etc. Without this formal relationship, the basis for accountability of providers towards enrolees may not be transparent. In fact, the literature suggests that the clearer the responsibilities of providers towards their enrolled population, the more there are gains in health outcomes induced by enrolment system [5]. It is also important to acknowledge that the features and potential benefits of an enrolment system are mutually reinforced by a capitation funding mechanism and the formal gatekeeping role of PHC, as well as dependant on the specific country context [5].

In Aotearoa New Zealand, the 2001 PHC Strategy (PHCS) [8] established Primary Health Organisations (PHOs)^3^ to provide essential PHC services to their enrolled populations. Having the full eligible population enrolled is essential for the PHCS to succeed, as stated at its inception [8], and reiterated in a later evaluation report [9]. This means that the desirable enrolment rate for the country is 100% of the eligible population. People usually enrol with a PHO through their usual GP. PHO enrolment is voluntary but encouraged by incentives for both practices and users. Enrolling allows people to benefit from lower consultation co-payments and reduced costs of prescription medicines [10]. For example, a GP consultation may cost around $50 for an enrolled adult, but around $100 if they are not enrolled [11–13]. Enrolled population also benefit from health promotion programmes via reminders for vaccination and cervical cancer testing, for example. In Aotearoa New Zealand, GP consultations are only free for the 0–14-year-old age group (since 2016) [14, 15]. GP consultations are financed mainly by a combination of out-of-pocket payments and capitation funding transfers [16]. Accurate enrolments are important for funding PHOs through the capitation formula, which pays a per capita rate for the enrolled population and relative need based on a PHO’s socio-demographic profile. This, in turn, supports equity in that funding is then available to support all of those who are enrolled, in contrast to a fee-for-service payment system that provides funding only for those using services. Providers then become responsible for the PHC of the population enrolled, and accountable through contracts. The contracts between PHOs and their District Health Boards (DHBs) detail both funding and specific targets for PHOs.

However, when not all the population is enrolled, the system may place some groups at a disadvantage, perpetuating inequities in health. International literature warns of the challenge of existing low and late enrolment coverage together with poor acceptability of services, particularly among specific population groups [5]. For example, if the capitation payments to practices for high need patients are perceived to be lower than the actual costs of delivering services to them, capitation may lead to ‘cream skimming’ of patients with higher needs, i.e. discouraging practices from enrolling high needs patients [4, 17]. A recent study in Ontario (Canada) found that enrolment rates in new comprehensive PHC models were consistently lower amongst immigrant groups than long-term residents, making it difficult to achieve equitable access to integrated PHC services for immigrant populations [18]. Consequently, the authors recommend ensuring enrolment by all population groups, taking into consideration social diversity, inequality and disadvantage to overcome low enrolment challenges by immigrant populations, such as outreach or drawing support from social networks [5]. In the case of Aotearoa New Zealand, data suggests that not all those eligible are enrolled, and that there are significant differences depending on population characteristics [19]. By analysing who is not enrolled with PHOs in Aotearoa New Zealand and how this has changed over time, we aim to achieve a better understanding of the equity implications of the enrolment system in PHC. Other countries employing patient registration systems in PHC may benefit from learning from the Aotearoa New Zealand case by drawing lessons that could be applied to improve their own enrolment systems and its monitoring.

Consequently, we investigate the extent and composition of populations enrolled in PHOs, addressing the following questions:

1. What proportion of people in Aotearoa New Zealand are enrolled in a PHO?
2. How did this change between 2015 and 2019?
3. What do we know about the socio-demographic profile of the population not enrolled?

## Methods

**Fig. 1:**
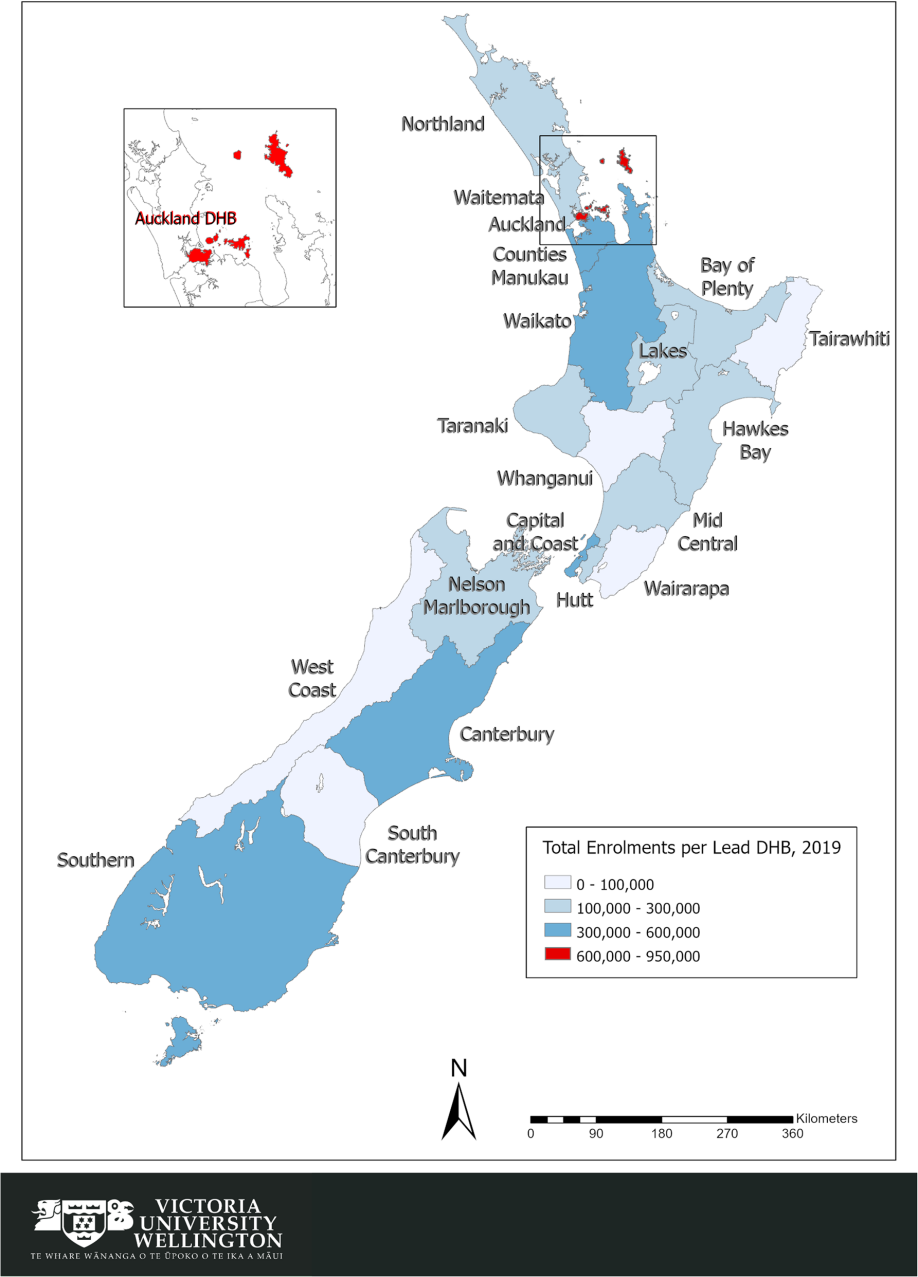
Aotearoa New Zealand Map - total PHO enrolments per DHB, January 2019. Source: Health Services Research Centre, Victoria University of Wellington (2019) from enrolment data from MoH (2019) [20]

There are twenty DHBs in Aotearoa New Zealand, responsible for providing or funding health services in their geographical districts. Figure 1 shows the map of Aotearoa New Zealand with all 20 DHBs and their enrolled population numbers, depicting the very different sizes of DHBs both in terms of enrolees as well as territory.

We analyse annual data on the proportion of people enrolled in a PHO. Data aggregated by District Health Boards (DHB) are updated quarterly at the Ministry of Health (MoH) website. Data are labelled ‘Access to Primary Care’ [21] and defined as per the formula:

$$ Enrolment\ rate=\left(\kern-0.3em \frac{No. of\ people\ enrolled}{Total\ population}\kern-0.3em \right) $$*100.

The complexity associated with this indicator is that there are two different sources of data for the numerator and denominator. The numerator comes from administrative data passed from PHOs to the Ministry of Health (MoH) through the National Enrolment Service (NES).^4^ The launch of a real-time NES system in 2015 served to better harmonize national data, although still there may be differences associated with progressive adoption of the NES^5^ and data collection by PHOs. There are limitations also from using the DHB of domicile, as an individual may choose to enrol with a PHO outside his/her DHB area [22], leading to a mismatch between numerator and denominator at DHB level. A person can only be enrolled in one PHO at a time; the system de-enrols patients when they enrol in a different PHO or three years following their last visit [23]. Each of these factors would affect reported enrolment rates.

The denominator is based on population projections from Statistics New Zealand [24] based on the 2013 Census, and provided in November 2017 for 2018 data, and in November 2018 for 2019 data.^6^ Although a new Census took place in 2018, delays with analysis and concerns around data quality [25, 26] have hindered its use. The potential misalignment between the two sources affects the accuracy of the indicator; for example, underestimation of population growth would lead to ‘false’ high enrolment rates, as has been identified as a concern in Counties Manukau DHB, a district with significant populations with low socioeconomic status [27].

We use annual enrolment rate aggregated data for all DHBs by ethnicity, age and deprivation for all available years at the time, 2015–2019, using fourth quarter data. The current presentation of data precludes analysis of different variables at the same time. The Aotearoa New Zealand population is made up of multiple ethnic groups, including: Māori, the indigenous populations of Aotearoa New Zealand, arriving in around the fourteenth Century from East Polynesia; those of European origin (New Zealand European), first settling in the country around the seventeenth Century; Pacific populations, whose origin is from the Pacific island nations; Asian groups (including Chinese and Indian), whose presence continues to grow and is expected to surpass Māori population by late 2020 [28]; and other groups. The data analysed classify ethnicity into three major groups: Māori, Pacific and New Zealand (NZ) European/Other, representing around 17, 8 and 75% of population respectively [29]. There is a likely mismatch in the ethnicity composition, between the prioritized ethnicity from the NES for the numerator, and from census prioritized data for the denominator [30]. The prioritization of ethnicity for analytical purposes (Māori, then Pacific, then NZ European/Other) means that those who identify as Māori and another ethnicity are categorized as Māori, so the NZ European/Other population is, more accurately, the non-Māori non-Pacific NZ European/Other population. As Pacific population enrolment rates often exceed 100% [21], due probably to inaccurate population projections, we present but do not discuss Pacific data in detail.

Deprivation classification is based on the New Zealand Deprivation Index 2013 (NZDep). It classifies each small geographical area according to its level of socioeconomic deprivation based on nine variables measured in the 2013 census. The resulting scores are ranked, then categorized into quintiles, where quintile 1 represents the least deprived areas and quintile 5 the most deprived ones [31]. Due to incomplete NZDep data, we adjust enrolment rates, when stratified by deprivation, by reducing the population numbers by an equal proportion across the five deprivation groups. The original and adjusted data are reported.

## Results

About 6% of the population was not enrolled in a PHO in 2019. Enrolment rates for the total population decreased from 95% in 2015 to 93% in 2018, recovering partially to 94% in 2019.

### PHO enrolment rates by ethnicity

**Fig. 2:**
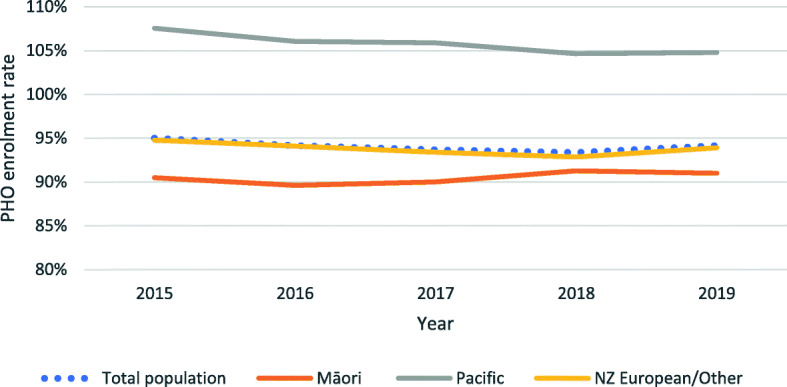
Evolution of percentage of population enrolled in a PHO, per ethnicity group, 2015–2019. Note: Y axis starts at 80%. Data source: Data compiled from MoH 2019 [21]

Māori have lower enrolment rates than the NZ European/Other group (91% compared to 94% in 2019) (Fig. 2). There is a slight closing of this gap during the period, due to improvements for Māori as well as decreasing rates for NZ European/Other. Pacific enrolment rates over 100% cannot be usefully interpreted due to data limitations.

The breakdown by DHBs reinforces that Māori have lower enrolment rates than the NZ European/Other population, as it is the case in all DHBs except for Hawkes Bay and Northland DHBs (see Table 1 in Online Appendix). The enrolment rate for Māori is particularly low in Auckland DHB (74%).

### PHO enrolment rates by age

**Fig. 3:**
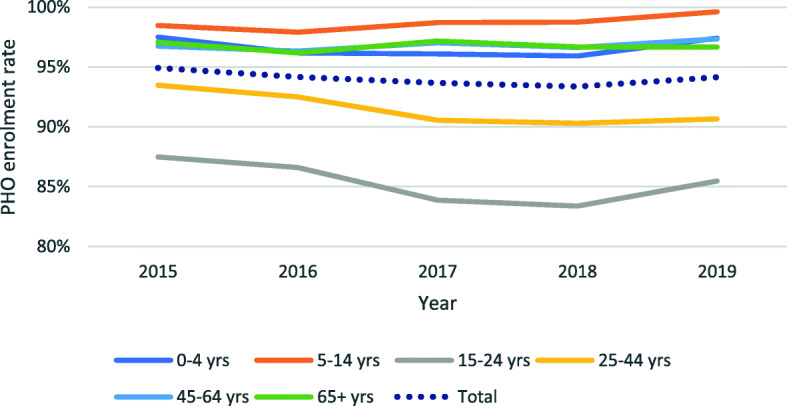
Evolution of percentage of population enrolled in a PHO, by age group, 2015–2019. Note: Y axis starts at 80%. The line for 45–65 years old group does not depict clearly as it largely overlaps with that of 65+ group. Data source: Data compiled from MoH 2019 [21]

It is young people (15–24 years old) followed by younger adults (25–44 years old) that experience the lowest enrolment rates (85 and 91% respectively in July 2019). These groups also have had the largest decrease between 2015 and 2019 (− 2 and − 3 percentage points respectively). The highest rates of enrolment are for children aged 5–14 years (100% in 2019). The differences across age groups widened over the study period (Fig. 3).

The differences between age groups are echoed in the results stratified by DHB. Most DHBs have the lowest rates of enrolment for the 15–24 years old group (see Table 2 in Online Appendix). Auckland DHB has the lowest rates for most age groups; one in three people aged 15–24 years in Auckland DHB is not enrolled.

### PHO enrolment rates by deprivation

**Fig. 4:**
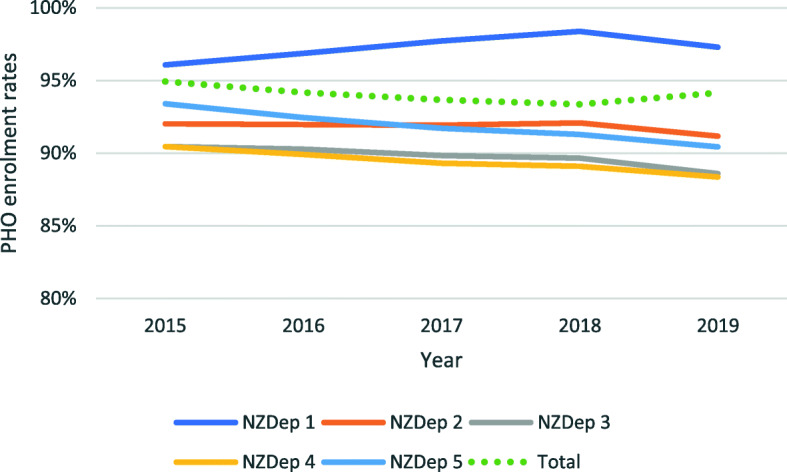
Evolution of percentage of population enrolled in a PHO, per deprivation quintile, 2015–2019. Note: Y axis starts at 80%. Data source: Data compiled from MoH 2019 [21]

The most affluent areas have the highest enrolment rates (97% for quintile NZDep 1) (Fig. 4) and enrolment rates have increased over time. Interestingly, national enrolment rates are lowest not for people living in the most deprived areas (NZDep 5), but for those in the middle-lower end (NZDep 4) (90 and 88% respectively in 2019). Data suggests that the differences in enrolment levels across deprivation quintiles have widened over time. A similar pattern was seen when enrolment rates were adjusted to account for missing NZDep data (those with no quintile assigned) (see Fig. 1 in Online Appendix).

The analysis by DHBs (Table 3 in Online Appendix) shows that enrolment rates for NZDep 1 exceed 100% for 14 DHBs in 2019, which suggests some problems with the data quality, most likely due to population projections from the census at a DHB level.

### PHO enrolment rates by DHB

The proportion of people enrolled in a PHO varies significantly across DHBs, ranging from 83% in Auckland DHB to 100% in Wairarapa and Northland DHBs (2019) (Table 1). Auckland DHB persistently has the lowest rates as well as the greatest decrease from 2015 to 2019 (− 8 percentage points), quite different from the majority of DHBs, as enrolment rates increased or remained similar for 14 DHBs over time.

**Table 1:** Variation of PHO enrolment rates across DHBs, 2015–2019

DHB of Domicile	2015	2016	2017	2018	2019	Change 2015–2019 (% points)
Auckland	91%	86%	84%	82%	83%	**−8**
Bay of Plenty	97%	99%	98%	99%	100%	**2**
Canterbury	94%	93%	93%	93%	93%	**−1**
Capital & Coast	94%	94%	94%	93%	93%	**−2**
Counties Manukau	99%	97%	97%	97%	97%	**−1**
Hawkes Bay	96%	97%	97%	97%	98%	**2**
Hutt Valley	97%	98%	98%	98%	98%	**1**
Lakes	99%	99%	98%	97%	98%	**−1**
Midcentral	93%	92%	93%	93%	94%	**1**
Nelson Marlborough	96%	96%	97%	97%	99%	**3**
Northland	98%	100%	100%	99%	99%	**1**
South Canterbury	98%	98%	97%	97%	98%	**0**
Southern	93%	92%	92%	92%	95%	**2**
Tairawhiti	97%	98%	98%	98%	98%	**1**
Taranaki	92%	93%	94%	94%	96%	**3**
Waikato	95%	95%	95%	94%	95%	**0**
Wairarapa	97%	99%	100%	101%	100%	**3**
Waitemata	94%	93%	92%	91%	92%	**−1**
West Coast	84%	92%	93%	94%	95%	**11**
Whanganui	97%	98%	99%	99%	99%	**2**
**NATIONAL**	**95%**	**94%**	**94%**	**93%**	**94%**	**−1**

Data source: Data compiled from MoH 2019 [21].

## Discussion

Our results show that a significant proportion of the population is not enrolled in any PHO, and this has slightly worsened since 2015. We observe striking inequities in PHC enrolment across sociodemographic groups and DHBs. Here we discuss potential explanations behind these trends.

First, data limitations emanate from using registered enrolments in the numerator and population projections in the denominator. Consequently, a 100% enrolment rate is unlikely to be achievable, as some people included in the ‘usually resident population’ are not eligible for publicly funded health services and therefore cannot enrol with PHOs [32]. Also, there may be people who are enrolled with a PHO but are not currently living in the country (i.e. counted in the numerator but not the denominator).

Second, as Auckland DHB comprises around 21% of national enrolments, it highly influences national rates. Enrolment rates in Auckland DHB are particularly susceptible to methodological issues given differences in enrolments when considering different definitions of DHB used in the data: DHB of domicile or lead DHB^7^: 457,278 vs 894,147 enrolees respectively in 2019 [19]. Some families may attend a GP in a location different to their domicile, resulting into enrolling in a different DHB; however, this does not seem to fully explain the low rates in Auckland DHB, as the other two neighbouring DHBs serving Auckland area - Counties Manukau and Waitemata DHBs - have rates below 100% and that are decreasing. Other factors for the low enrolment rates may relate to Auckland having a higher proportion of young people, a growing population through internal migration^8^ and a potential higher proportion of residents not qualifying for New Zealand public funding for health care. These factors may contribute to underestimation of real enrolment rates.

Beyond data and socio-demographic factors, there seem to be issues suggesting worsening in PHC access. Some practices are not taking new enrolees as they reach their full capacity, in what is called ‘closed books’. About 11% of GPs reported that their practices did not accept new enrolments in 2018, up from 10% in 2017, and it is even higher (17%) for practices identified as not clearly urban or rural (2018) [33], which suggests we need to identify the profile of population served by these practices. This relates to the recognised decreasing rate of GPs per population, and further shortages of GPs are expected as GPs reach retirement age [34]. Current and expected future GP shortages are a global issue, also reported in Australia [35, 36] and the UK [37] for example. The issue of closed books would suggest that people are willing but unable to enrol. Another factor may be the possibility of people not enrolling because of legal or immigration concerns, as these are one of the recognised barriers to accessing care by migrant populations worldwide [38].

The lower enrolment rates for youth and younger adults may seem reasonable given that people aged 15–24 are generally healthy and have a lower level of need for medical care. Yet, the New Zealand Health Survey (NZHS) reveals that about 24% of the 15–24 years old and 26% of the 25–44 years old groups recognise unmet need for PHC, compared to 18% by those 65+ years (pooled 2014–2017 data) [39]. Higher population mobility for the 15–24 years group may be another factor, but the low rate for young people in Auckland DHB does not seem to be substantiated by over-enrolments in other DHBs. Another explanation is that young people tend to have lower incomes and consultation fees may deter them from visiting a GP, even with the lower fees for those aged 14–17. The low enrolment rates of young people suggest the need to monitor that their needs are being met. Promoting young people’s enrolment in PHOs may be a way to support their connection with health services. Worldwide, it is recognised [40] that adolescents and youth face specific challenges in accessing health services around issues of staff attitudes, age-appropriate environment, etc., and that promoting youth and adolescents-friendly health services is key to enhance their utilization by young populations. In addition, Zeratsion [41] advocates raising the age threshold for zero PHC fees based on the experience in Norway of increases in PHC use among adolescents following co-payment exemptions, and that late adolescents would have more health needs that younger ones.

We would expect newly born children be enrolled and remain so in their early years given that standard consultations are free for young children, their needs for a greater intensity of visits for follow ups and vaccinations, and because providers can pre-enrol new-borns before their full enrolment process is completed [42]. Enrolment rates like 88% in West Coast DHB or 92% in Capital Coast DHB suggest potential data issues such as a potential higher birth rate projected in these two DHBs that would lead to underestimation of real enrolment rates. Children aged 5–14 years have the highest enrolment rates attained for any age group, above 97% for nearly all DHBs. This backs up the positive effect of zero fees on enhancing access to health services for children. Moreover, if it is possible to achieve such high rates for those aged 5–14, it may be possible for other age groups also. A suggestion would be using these highest enrolment rates for children aged 5–14 as the benchmark for the rest of age groups to achieve, perhaps a more realistic target than 100%.

In relation to ethnicity, not only are Māori less likely to be enrolled than NZ European/Other population nationally (91% vs 94% in 2019), but this disparity persists throughout the period and it is reflected by all but two DHBs in 2019. This difference may be partly explained by the Māori population being younger. Other factors include barriers to accessing PHC services, such as financial, transport and child care costs; Māori children and adults are more likely than non-Māori to have unmet need and unfilled prescriptions [43]; in 2016/17, the prevalence of one or more types of unmet need^9^ reported by Māori adults was 38%, compared to 28% for non-Māori [43]; and about 22% of Māori aged 15+ years had unmet need for GP services due to cost in the last year, compared to 13% for NZ European/Other [44, 45]. Nonetheless, the ethnicity breakdown is particularly susceptible to inconsistencies given that the percentage of people identified as Māori is likely to be higher in the numerator than the denominator, as argued by Chan et al. [30]

The deprivation analysis points at the widening gap between deprivation quintiles. We have identified a data caveat here,, the contradictory trends between specific quintiles and the overall national picture (Fig. 4). These may be due to the exclusion from the quintile breakdown of those not having a deprivation level assigned, but who are included in the national rate and population estimations^10^; this problem may be more accentuated for year 2019 when automatic NES enrolment was fully functional and the proportion of people with missing NZDep scores went from 1.22% (2018) to 2.94% (2019). Our attempt to align enrolments with national figures helps to understand the time trends, though it remains imprecise in itself; missing data is probably not random, so applying it equally to the five groups introduces a bias, just as missing data does. Further work is required to better understand the relationship between PHO enrolments and deprivation levels. All in all, the fact that enrolment rates are higher for the most deprived deciles than for those in the middle-lower end suggests the positive impact of targeted schemes in enhancing access of the most deprived areas. Still, NZHS suggests that unmet need for PHC remains highest for the most deprived quintile (34%) and fairly constant between 2011 and 2017, compared to 23% for the least deprived quintile.

The results and implications discussed point at the need to consider additional ways to ensure specific groups of people do enrol, such as outreach or drawing support from social networks [5]. Our findings are consistent with international literature [4, 5, 18] in that PHC enrolment rates serve to identify inequities in health care access. Efforts should be made to reduce methodological flaws of the indicator as recommended in our deprivation analysis and more broadly by Chan et al. [30] We recommend investigating differences in health outcomes between those enrolled and not enrolled, which could be guided by similar analysis in Ontario, Canada [18, 46] and related research in New Zealand [47]. We also suggest examining the relationship between enrolment data with other variables such as continuity of care and other health care usage (emergency department, hospitalization), to identify more accurately the population who is or not ‘attached’ to their primary care provider, as done in Canada [46]. Further analysis could also include understanding how promoting PHO enrolment may enhance continuity of care. Individual-level enrolment data would render more detail and allow to control for the effects of multiple variables. A multi-country comparison of enrolment rates could render a better understanding of for example targets for specific age or deprivation groups. Finally, our understanding of how an enrolment system may serve to promote equity in health care would benefit from considering a wider understanding of equity in health that takes into account acceptability [48], including the perceptions of potential users around equity and access as recommended by Mooney [49]. This could be particularly relevant for Māori and young and adolescent potential users [48].

## Conclusion

Having as many people as possible enrolled with a PHC provider is instrumental to maximising the full potential of PHC for all, and to enhancing equitable access to services. In Aotearoa New Zealand, not all eligible people are enrolled with a PHO and inequities exist between socio-demographic and geographic groups. We have discussed potential reasons behind the enrolment gap, both methodological inconsistencies as well as reasons pointing at worsening access to health care and inequities. We need to better understand why people are not enrolling with a PHO and its implications. We suggest using more intensively PHO enrolment rates as a proxy to monitor PHC access and equity and to track progress towards achieving the goals of the PHCS. In particular, reducing disparities across population groups could be used to track equity in health access. We need to understand the reasons behind the potential enrolment gap and widening of inequities identified, in particular the lower enrolments of Māori and young populations and widening gaps across deprivation areas.

The research deepens our understanding of the enrolment rates and its potential pitfalls specially in relation to equity in the case of Aotearoa New Zealand. These recommendations apply not only for Aotearoa New Zealand but for all countries using patient enrolment systems. These countries can learn from Aotearoa New Zealand’s differences in enrolments across districts, age, ethnicity and deprivation levels to draw lessons for improving equity in health care. It is important to tease out and monitor over time the extent and composition of the populations not being enrolled and the reasons and implications for health access and outcomes in order to keep track of equity. These equity concerns need to be considered when adapting PHC enrolment systems and associated funding models across countries.

The underlying motivation of this study is that we need good proxies to monitor the performance of PHC services; having the highest possible proportion of the population enrolled is the starting point to examine both performance and equity in a system using patient enrolments. For countries with this system, having more robust indicators and data around the enrolment gap will provide more precise and evidence-based understanding of the equity challenges in PHC. This in turn will enable prioritizing the redress of inequities in health policy. PHC advocates need to promote improving these indicators and their use as a starting point to monitor and prioritize equity in health care.

## Supplementary Information

**Additional file 1 Table S1.** Percentage of population enrolled in a PHO, per ethnicity groups and DHBs, 2019. **Table S2.** Percentage of population enrolled in a PHO for age groups and DHBs, 2019. **Table S3.** Percentage of population enrolled in a PHO, per deprivation group (NZDep) and DHBs, 2019. **Figure S1.** Evolution of percentage of population enrolled in a PHO, per adjusted* deprivation quintiles, 2015-2019.

## Data Availability

the datasets analysed in the current study are publicly available from New Zealand Ministry of Health (https://www.health.govt.nz/our-work/primary-health-care/about-primary-health-organisations/enrolment-primary-health-organisation).

## Abbreviations

DHB: District Health Board
GP: General Practitioner
MoH: Ministry of Health (New Zealand)
NES: National Enrolment System
NZ: New Zealand
NZDep: New Zealand Deprivation Index
NZHS: New Zealand Health Survey
OECD: Organisation for Economic Co-operation and Development
PHC: Primary Health Care
PHCS: Primary Health Care Strategy (New Zealand, 2001)
PHO: Primary Health Organization

## Footnotes

1 Australia, Canada, Chile, Estonia, Finland, Hungary, Ireland, Israel, Italy, the Netherlands, New Zealand, Norway, Poland, Portugal, Slovenia, Spain Sweden, the United Kingdom

2 , Belgium, Denmark, France, Iceland, Mexico, Latvia, Lithuania, the Slovak Republic, Switzerland, the United States

3 PHOs are local, non-governmental, not-for- profit organisations, funded by their local District Health Boards to manage primary health services for their enrolled populations [8].

4 The ENS is an IT enhanced system that enables real-time patient enrolment status data from all GPs and centralised It allows calculations for capitation funding based on updated enrolled users. It also contains National Health Index (NHI) and patient demographics, which allows research into how demographic groups portray in different practices/PHOs/DHBs that inform equity analysis [23].

5 Personal communication with Ministry of Health, Primary Care Department, 28 February 2020

6 This suggests that population projections are updated annually from Statistic New Zealand, though there are no notations for 2015–2017 data to confirm this.

7 There are two categories of DHB used in health statistics in Aotearoa New Zealand: ‘DHB of domicile’ - based on the address of the enrolee, and ‘lead DHB’ - used for capitation funding transfers. A PHO can have agreements with several DHBs but receives all its funding from the lead DHB.

8 Assuming population projections account for these population movements and with a time lag in enrolling with a GP in a new location.

9 Unmet need for primary health care indicates whether people experience any of the following barriers to accessing primary health care in the past 12 months: Unmet need for a GP due to cost; Unmet need for an after-hours medical centre due to cost; Unmet need for a GP due to lack of transport; Unmet need for an after-hours medical centre due to lack of transport; Inability to get an appointment at their usual medical centre within 24 h [39].

10 Personal communication with Ministry of Health, Primary Care Department, 28 February 2020.
