# Supplementary material for: The enrolment gap: who is not enrolling with primary health organizations in Aotearoa New Zealand and what are the implications? An exploration of 2015–2019 administrative data
Source: Int J Equity Health. 2021 Apr 6;20:93. doi: 10.1186/s12939-021-01423-4 (PMC8025352; doi:10.1186/s12939-021-01423-4)
Supplement: Supplementary file 1 — Additional file 1 Table S1. Percentage of population enrolled in a PHO, per ethnicity groups and DHBs, 2019. Table S2. Percentage of population enrolled in a PHO for age groups and DHBs, 2019. Table S3. Percentage of population enrolled in a PHO, per deprivation group (NZDep) and DHBs, 2019. Figure S1. Evolution of percentage of population enrolled in a PHO, per adjusted* deprivation quintiles, 2015-2019. [file 12939_2021_1423_MOESM1_ESM.docx]

**Online Appendix**

**Table 1: Percentage of population enrolled in a PHO, per ethnicity groups and DHBs, 2019.**

| **DHB OF DOMICILE** | **MĀORI** | **PACIFIC** | **NZ EUROPEAN/OTHER** | **TOTAL** |
| --- | --- | --- | --- | --- |
| Auckland | 74% | 98% | 82% | 83% |
| Bay of Plenty | 98% | 88% | 100% | 100% |
| Canterbury | 86% | 108% | 93% | 93% |
| Capital & Coast | 88% | 100% | 93% | 93% |
| Counties Manukau | 92% | 116% | 93% | 97% |
| Hawkes Bay | 99% | 93% | 98% | 98% |
| Hutt Valley | 92% | 99% | 100% | 98% |
| Lakes | 97% | 101% | 98% | 98% |
| Mid Central | 83% | 93% | 97% | 94% |
| Nelson Marlborough | 89% | 88% | 100% | 99% |
| Northland | 104% | 88% | 98% | 99% |
| South Canterbury | 84% | 141% | 99% | 98% |
| Southern | 87% | 102% | 95% | 95% |
| Tairawhiti | 98% | 79% | 99% | 98% |
| Taranaki | 87% | 97% | 98% | 96% |
| Waikato | 91% | 93% | 97% | 95% |
| Wairarapa | 99% | 103% | 100% | 100% |
| Waitemata | 82% | 99% | 93% | 92% |
| West Coast | 87% | 79% | 96% | 95% |
| Whanganui | 97% | 99% | 100% | 99% |
| **NATIONAL** | **91%** | **105%** | **94%** | **94%** |

Data source: Data compiled from MoH 2019^14^.

**Table 2: Percentage of population enrolled in a PHO for age groups and DHBs, 2019**

| **DHB OF DOMICILE** | **0-4 YRS** | **5-14 YRS** | **15-24 YRS** | **25-44 YRS** |
| --- | --- | --- | --- | --- |
| Auckland | 95% | 96% | 67% | 74% |
| Bay of Plenty | 99% | 103% | 94% | 96% |
| Canterbury | 95% | 97% | 85% | 92% |
| Capital & Coast | 92% | 98% | 83% | 91% |
| Counties Manukau | 101% | 106% | 92% | 94% |
| Hawkes Bay | 99% | 101% | 98% | 100% |
| Hutt Valley | 100% | 101% | 95% | 101% |
| Lakes | 100% | 102% | 90% | 97% |
| Mid Central | 99% | 100% | 82% | 90% |
| Nelson Marlborough | 99% | 99% | 99% | 98% |
| Northland | 97% | 99% | 96% | 98% |
| South Canterbury | 93% | 97% | 101% | 104% |
| Southern | 97% | 98% | 82% | 95% |
| Tairawhiti | 97% | 97% | 95% | 98% |
| Taranaki | 99% | 97% | 94% | 92% |
| Waikato | 98% | 99% | 88% | 93% |
| Wairarapa | 101% | 99% | 101% | 97% |
| Waitemata | 97% | 100% | 83% | 89% |
| West Coast | 88% | 87% | 92% | 91% |
| Whanganui | 97% | 100% | 98% | 95% |
| **NATIONAL** | **97%** | **100%** | **85%** | **91%** |

Data source: Data compiled from MoH 2019^14^.

**Table 3: Percentage of population enrolled in a PHO, per deprivation group (NZDep) and DHBs, 2019.**

| **DHB OF DOMICILE** | **NZ DEP 1 - 2** | **NZ DEP 3 - 4** | **NZ DEP 5 - 6** | **NZ DEP 7 - 8** |
| --- | --- | --- | --- | --- |
| Auckland | 84% | 81% | 80% | 79% |
| Bay of Plenty | 116% | 97% | 99% | 93% |
| Canterbury | 102% | 89% | 83% | 81% |
| Capital & Coast | 95% | 92% | 88% | 83% |
| Counties Manukau | 93% | 93% | 90% | 95% |
| Hawkes Bay | 102% | 96% | 92% | 93% |
| Hutt Valley | 99% | 99% | 94% | 96% |
| Lakes | 100% | 93% | 89% | 91% |
| Mid Central | 99% | 95% | 88% | 88% |
| Nelson Marlborough | 104% | 94% | 93% | 91% |
| Northland | 102% | 98% | 95% | 97% |
| South Canterbury | 102% | 97% | 93% | 93% |
| Southern | 105% | 92% | 89% | 86% |
| Tairawhiti | 112% | 98% | 95% | 94% |
| Taranaki | 105% | 92% | 85% | 84% |
| Waikato | 104% | 92% | 91% | 89% |
| Wairarapa | 103% | 101% | 100% | 96% |
| Waitemata | 93% | 91% | 89% | 88% |
| West Coast | 112% | 92% | 86% | 86% |
| Whanganui | 100% | 97% | 91% | 95% |
| **NATIONAL** | **97%** | **91%** | **89%** | **88%** |

Data source: Data compiled from MoH 2019^14^.

**Figure 4: Evolution of percentage of population enrolled in a PHO, per adjusted* deprivation quintiles, 2015-2019.**

*Based on an artificial weighted national average from five deprivation quintiles

Note: The NZDep 0, those with no deprivation data, have been assigned on equal basis across the five deprivation groups to account for all population in both numerator and denominator.

Note: Y axis starts at 80%.

Data source: Data modified from MoH 2019^14^.
